# Mitochondrial DNA sequencing demonstrates clonality of peritoneal implants of borderline ovarian tumors

**DOI:** 10.1186/s12943-017-0614-y

**Published:** 2017-02-27

**Authors:** Giulia Girolimetti, Pierandrea De Iaco, Martina Procaccini, Riccardo Panzacchi, Ivana Kurelac, Laura Benedetta Amato, Giulia Dondi, Giacomo Caprara, Claudio Ceccarelli, Donatella Santini, Anna Maria Porcelli, Anna Myriam Perrone, Giuseppe Gasparre

**Affiliations:** 1 Department of Medical and Surgical Sciences (DIMEC) - Unit of Medical Genetics, University of Bologna Medical School, Via G. Massarenti 9, 40138 Bologna, Italy; 2 Department of Obstetrics and Gynecology, Oncologic Gynecology Unit, University Hospital S.Orsola-Malpighi, 40138 Bologna, Italy; 3 Unit of Pathology, University Hospital S.Orsola-Malpighi, 40138 Bologna, Italy; 4 Unit of Oncology and Transplant Pathology, University Hospital S.Orsola-Malpighi, 40138 Bologna, Italy; 5 Department of Experimental, Diagnostic, and Specialty Medicine, University Hospital S.Orsola-Malpighi, 40138 Bologna, Italy; 6 Department of Pharmacy and Biotechnology, University of Bologna, 40138 Bologna, Italy

**Keywords:** Gynecological cancer, Mitochondrial DNA mutations, Borderline ovarian tumors, Peritoneal implants

## Abstract

Borderline ovarian tumors are rare low malignant potential neoplasms characterized by the absence of stromal invasion, whose main prognostic factors are stage and type of peritoneal implants. The latter are defined as invasive when cell proliferation invades the underlying tissue (peritoneal surface, omentum and intestinal wall), or noninvasive. It is still unknown if these implants are metastatic spread from the primary ovarian mass or a neoplastic transformation *de novo* of the peritoneal surface. Mitochondrial DNA sequencing was performed to assess clonality in eight patients presenting both borderline ovarian tumors and implants. In 37.5% of the cases, the same mitochondrial DNA mutation was present in both borderline ovarian tumors and the peritoneal implant, being this evidence that implants may arise as a consequence of a spread from a single ovarian site.

**Electronic supplementary material:**

The online version of this article (doi:10.1186/s12943-017-0614-y) contains supplementary material, which is available to authorized users.

## Findings

### Background

Borderline ovarian tumors (BOTs) represent a heterogeneous group of noninvasive neoplasms of low malignant potential. They comprise about 15–20% of all epithelial ovarian malignancies [1] and are usually diagnosed as being limited to the ovary. The 10-year survival rate for women with stage I BOT is around 70%, caused by subsequently recurrent disease or progression to invasive carcinoma [2, 3]. Standard treatment is hysterectomy with bilateral adnexectomy and multiple peritoneal biopsies. In young patients conservative treatment is an option and they may undergo surgery limited to a unilateral salpingo-ovariectomy with multiple biopsies [4]. Serous histotype represents 65% of all the BOTs [5], about 35% of which can occur in association with serous lesions involving the peritoneum, i.e. implants, defined as either invasive or noninvasive depending on their microscopic appearance. Invasive implants are found in a lower number of patients (22%) compared to noninvasive ones (78%) [5], and the survival rate is around 66% after a mean follow-up of 7.4 years, compared to 95% for patients with noninvasive implants [6]. A study on 80 cases of serous BOTs with noninvasive implants showed that after a follow-up of 15 years, 44% of patients presented recurrences and 25% died of disease [7]. The pathological stage and sub-classification of extra-ovarian disease into invasive and noninvasive implants, together with the presence of postoperative macroscopic residual masses, currently appears to be the major predictor not only for recurrence, but also for survival [8, 9].

In the last years, clonality studies have attempted to elucidate whether multiple tumor nodules arise as a result of a spread from a single ovarian site or whether such deposits are polyclonal, representing independent primary tumors, with discordant results [10, 11].

BOT patients recurrence and survival change substantially depending on whether a peritoneal implant arises or not. Since women with BOT and peritoneal lesions usually have a good prognosis, the latter are classified as implants instead of metastases. These masses are considered as an extra-ovarian spread of the primary tumor [11], but several studies highlight their differences with the latter, considering implants as independent masses of polyclonal origin [10]. In spite of this still open dilemma, approaches for discrimination between the monoclonal or polyclonal nature of peritoneal implants are still lacking. Mitochondrial DNA sequencing was recently shown to be a robust tool to define clonality in simultaneously detected tumors of the female genital tract [12, 13] as it is virtually impossible that the same tumor-specific mutation may arise in two independent neoplasms. We here apply such technique to BOTs and their implants.

## Results

We performed whole mitochondrial DNA sequencing on eight patients presenting with serous BOTs and implants (Table 1), after collection of informed consent within the frame of the Mitochondria in Progression of Endometrial and Ovarian cancer - MiPEO study, approved by the local ethical committee. An alpha-numeric code (from B1 to B8) was assigned to the cases to maintain anonymity.

**Table 1:** Cases and histopathology

Patients	Age at diagnosis	Surgery	Histology	Staging (FIGO 2014)	Implant type	Implant localization	DFS	OS
B1	35	FS	Serous BOT	IIB	Non invasive	Pelvic peritoneum	48	74
B2	44	CS	Serous BOT	IIC3	Non invasive	Pelvic peritoneum	34	34
B3	30	FS	Serous BOT with intraepithelial carcinoma	IIIA	Non invasive	Pelvic peritoneum and omentum	31	31
B4	81	CS	Serous BOT	IIB	Non invasive	Pelvic peritoneum	120	120
B5	23	FS	Serous BOT with intraepithelial carcinoma	IIC3	Invasive	Pelvic peritoneum	30	67
B6	32	FS	Serous BOT	IIC3	Non invasive	Pelvic peritoneum	7	68
B7	34	FS	Serous BOT	IIIA	Non invasive	Pelvic peritoneum, pararectal peritoneum	13	128
B8	63	CS	Serous BOT	IIIA	Non invasive	Pelvic peritoneum, omentum and right diaphragmatic peritoneum	16	16

Age, surgery, histology and FIGO stage of borderline ovarian tumors, implants type and localization, months from treatment until first relapse and months from diagnosis to last follow up are shown

*Abbreviations*: *FS* Fertility Sparing treatment, *CS* Complete Staging, *BOT* Borderline Ovarian Tumor, *DFS* Disease Free Survival, *OS* Overall Survival

Three out of eight patients underwent complete staging and 5/8 (62.5%) fertility sparing treatment following their wish to become pregnant. Based on histopathological analyses, all samples were serous BOTs, 2 of whom with one small focus of intraepithelial cancer in the ovarian cyst. Noninvasive implants were diagnosed in 7/8 cases (87.5%), only one patient presenting with an invasive implant (12.5%), and relapsing after 30 months from the first surgery. Recurrences were observed only in patients with fertility sparing treatment (4/5–80%). Three out of seven patients (42.8%) diagnosed with noninvasive implants presented relapse after their first surgery (Table 1). All patients were alive and free of disease at the latest follow up.

The entire mitochondrial DNA sequence was obtained from all 16 single BOT and implant samples [14] and variants carefully filtered for pathogenicity through MToolBox [15, 16]. Detailed materials and methods are available as Additional file 1. For all eight patients, DNA extracted from unaffected tissue was used to detect tumor-specific and non-tumor-specific variants. Sequences of B1-B8 samples, including matched non-tumor sequences, were submitted to the public human mitochondrial database HmtDB [17] and a list of specimens and HmtDB identifiers is reported in Additional file 2. Overall, 7 tumor-specific variants in coding genes were found in 4/8 patients (50%) (Table 2).

**Table 2:** Mitochondrial DNA mutations

Sample	Mitochondrial DNA Mutations	Mitochondrial DNA mutations localization	Mutation type	Amino Acid substitution	Gene	Variability	DS
B1	m.4810G >A	BOT	Nonsense	W114X	*MT-ND2*	0.0	-
	m.15570T >C	BOT + PI	Missense	L275P	*MT-CYB*	0.0	0.892
B2	m.3428G >A	BOT	Missense	G41D	*MT-ND1*	0.0	0.909
	m.15219insA	BOT	Frameshift	-	*MT-CYB*	0.0	-
B3	m.11984T >C	BOT + PI	Missense	Y409H	*MT-ND4*	0.002	0.764
B4	m.3352G >A	BOT	Missense	A16T	*MT-ND1*	0.0	0.827
	m.15449T >C	BOT + PI	Missense	F235L	*MT-CYB*	0.00642	0.088
	m.16189T >C	BOT + PI	SNP	-	*MT-D-loop*	0.767	-
B5	m.310insC	BOT	Insertion	-	*MT-D-loop*	0.215	-
	m.310delCC	PI	Deletion	-	*MT-D-loop*	0.215	-

All mitochondrial DNA mutations reported in the table are tumor-specific and heteroplasmic

*Abbreviations*: *BOT* Borderline Ovarian Tumor, *PI* Peritoneal Implant, *SNP* Single Nucleotide Polymorphism, *DS* Disease Score

**Fig. 1:**
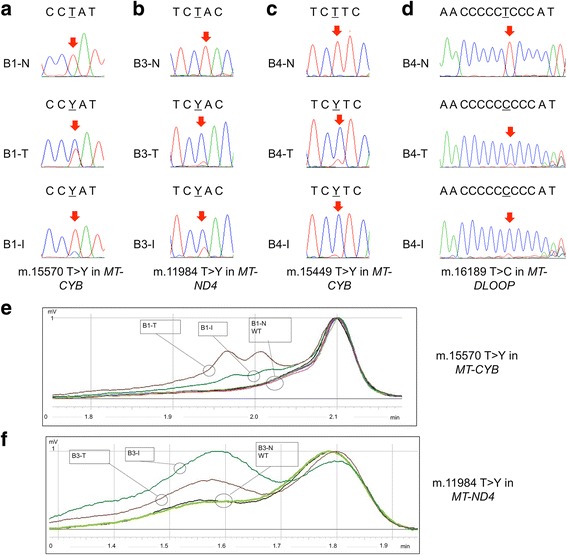
Mitochondrial DNA sequencing in BOTs and implants (**a**, **b**, **c**, **d**). Electropherograms of mitochondrial loci harboring mutations in BOT and peritoneal implant samples. Red arrows indicate the mutated bases. (**e**, **f**) Representative dHPLC elution profiles for the in-depth investigation of the somatic nature of mitochondrial DNA mutations m.15570 T > Y/*MT-CYB* in case B1 and m.11984 T > Y/*MT-ND4* in case B3. Homo- and hetero-duplexes are distinguished based on different retention times. (**e**) Two elution curves for T (Tumor) and I (Implant) (heteroduplex and homoduplex) and a single elution curve for non-tumor tissue (N) and three wild-type controls are present in the analysis of m.15570 T > Y/*MT-CYB* in case B1. Wild-type (*black, pink and purple*), N (*light green*), I (*green*), T (*brown*). (**f**) Two elution curves for T and I (heteroduplex and homoduplex) and a single elution curve for non-tumor tissue (N) and one wild-type control are present in the analysis of m.11984 T > Y/*MT-ND4* in case B3. Wild-type (*black*), N (*light green*), I (*green*), T (*brown*)

In patient B1, the m.15570T > C/(*MT-CYB*) was found in both BOT and implant (Fig. 1a) whereas the m.4810G > A/(*MT-ND2*) was found only in BOT. B2 carried the mutations m.3428G > A/(*MT-ND1)* and m.15219insA/(*MT-CYB*) only in BOT. In patient B3, we found the m.11984 T > C/(*MT-ND4*) in both BOT and implant (Fig. 1b). In patient B4, the m.15449 T > C/(*MT-CYB*) was found in both BOT and implant (Fig. 1c) whereas the m.3352G > A/(*MT-ND1*) was found only in BOT. None of the corresponding matched non-tumor samples was shown to carry these mitochondrial DNA mutations. All mitochondrial DNA mutations found were heteroplasmic in the tissues, although the mutation load may be in such cases underestimated, due to a potential contamination by non-tumor cells, whose complete exclusion from dissected tissues is virtually impossible.

All variants featured a low variability value and, in 4/5 (80%) cases, a high score of disease indicating possible candidate variants affecting function (Table 2) [16].

To establish the tumor specificity of the mutations found in both BOTs and implants of the samples B1, B3 and B4 and to confirm a diagnostic efficiency of mitochondrial DNA sequencing we performed dHPLC, which is able to detect heteroplasmic mutations as low as 2% [18]. Low-level germline heteroplasmy was absent from non-tumor tissue of case B1, in which the m.15570 T > C/(*MT-CYB*) was shown to be present exclusively in BOT and implant samples (Fig. 1e) and absent from non-tumor tissue. We performed the same analysis for cases B3 (Fig. 1f) and B4 (data not shown) revealing that the m.11984 T > C/(*MT-ND4*) and the m. 15449 T > C/(MT-CYB) were present exclusively in BOT and implant samples and absent from non-tumor tissues. According to these data, the germline nature of mutations found in both BOTs and implant samples of B1, B3 and B4 was ruled out, allowing to conclude that tumor-specific mitochondrial DNA mutations in coding genes were detected in 50% (4/8) of BOT at different heteroplasmy levels. In 37.5% (3/8) of the cases, the same mitochondrial DNA mutation was present in both BOT and the peritoneal implant. The extremely low variability of the informative mutations found (Table 2) strengthens the clonality hypothesis, as they are unlikely to occur independently in different cells, as the current variability estimates for mitochondrial genome positions show that a great part of over 16500 nucleotides of the human mitochondrial DNA varies among individuals with different frequency, as it is reported in HmtDB [17]. It is worth underlining that somatic mutations that were found in our sample set, exclusively in the BOT and were not shared with the peritoneal implant, by no means rule out a clonal origin of the two neoplasms. Mitochondrial DNA variants found only in BOTs may indeed be subsequent to the initial clonal expansion, especially in view of their heteroplasmic nature. It is of note that all somatic mutations in the coding sequences presented a heteroplasmic status in the mass and a very low variability value. They may hence be still unfixed events, particularly since they represent highly damaging genetic lesions. It is known that a certain degree of mitochondrial respiratory chain activity needs to be maintained to progress towards malignancy, and accumulation of damaging mitochondrial DNA mutations blunts tumorigenesis [19]. The occurrence of heteroplasmic mutations may therefore be explained by the need for a metabolic adaptation and by the advantages they confer through the enhancement of reactive oxygen species production associated with tumor promotion [20, 21].

Interestingly, patients B1, B3, B4, and B6 were diagnosed with serous BOTs with noninvasive implants. In sample B3, a small area of low grade intraepithelial carcinoma was found. Patient B1 presented a relapse after the first surgery. Mitochondrial DNA mutations may, in this context, concur to foster transformation of borderline tumor cells into a more aggressive and invasive type of cancer, making their use two-fold both in determining clonality, hence allowing to identify metastases, and to potentially infer a clinical behavior, thus aiding to delineate the prognosis.

We last focused on the variants mapping in the D-loop fragile spots (long C-traits), as these have been already proposed as a marker for clonality [22, 23]. A high frequency of the TC insertion at nucleotide position 310 was found in early stages of serous BOTs [24]. Indeed, we found that the only positions with variants were around nucleotides 303–309, a fragile poly-C stretch termed D310, and 16189, as expected. Inspection of D310 revealed that in patient B6 a heteroplasmic insertion of a cytosine was present only in BOT and peritoneal implant, although Fluorescent PCR [19] revealed a very low load of the insertion in the matched non-tumor sample, thereby blunting the informative potential in this case. Sample B5 carried a heteroplasmic insertion of a cytosine in the BOT tissue while peritoneal implant carried the heteroplasmic deletion of two cytosines. The matched non-tumor sample was shown to carry no insertions or deletions. Concerning the analysis of the mitochondrial 16189 variant, characterized by a T > C substitution, which produces a highly variable and fragile poly-C tract, we found the T > C substitution in BOT and implant tissue of patient B4, not occurring in the matched non-tumoral tissue (Fig. 1d), confirming the BOT and the peritoneal implant to have the same origin.

Overall, tumor-specific mitochondrial DNA mutations were detected in 62.5% (5/8) of BOT at different heteroplasmy levels. In 37.5% of the patients, the same mitochondrial DNA mutation was present in both the BOT and the peritoneal implant and was therefore informative to infer clonality.

## Conclusions

Besides reporting here for the first time the occurrence of pathogenic mitochondrial DNA mutations in BOTs, our findings have relevant implications in the patient management. Distinguishing between a polyclonal and a monoclonal origin of implants is pivotal in deciding therapeutic options. Indeed, for implants that do not originate from the primary tumor a peritoneal carcinogenesis may be envisioned as a potential cause, likely to have occurred within the Müllerian islands, whereby a synchronous tumor to the primary BOT may develop. In this case, removal of the primary BOT does not protect from the formation of implants, implying that a surgical exploration of all peritoneal surfaces and multiple peritoneal biopsies should be mandatory and radical surgery on reproductive organs such as contralateral ovary and uterus plays a secondary role. On the other hand, the demonstration that implants are of a monoclonal derivation implies that they ought to originate from a plundering of the primary tumor. Therefore, in such cases, early diagnosis and removal of the primary tumor becomes pivotal in the prevention of spread within the abdominal cavity, accounting for the importance of molecular analyses capable of providing such a relevant proof of concept.

## Additional files

Additional file 1: Supplementary materials and methods. Detailed Materials and Methods are reported. (DOCX 32 kb)

Additional file 2: Sample IDs of Borderline Ovarian Tumors, Peritoneal Implants and Non-Tumor tissue. Sequences of B1-B8 samples were submitted to the public database HmtDB (http://www.hmtdb.uniba.it), list of specimens and HmtDB identifiers are reported. Abbreviations: Borderline ovarian tumors (B); peritoneal implants (I); non-tumor tissue (N). (XLSX 9 kb)

## Abbreviations

BOT: Borderline ovarian tumors
dHPLC: Denaturing High Performance Liquid Chromatography
