# Supplementary material for: Mitochondrial DNA sequencing demonstrates clonality of peritoneal implants of borderline ovarian tumors
Source: Mol Cancer. 2017 Feb 27;16:47. doi: 10.1186/s12943-017-0614-y (PMC5327524; doi:10.1186/s12943-017-0614-y)
Supplement: Supplementary file 1 — Supplementary materials and methods. Detailed Materials and Methods are reported. (DOCX 32 kb) [file 12943_2017_614_MOESM1_ESM.docx]

Material and Methods

Case Series

This study was conducted within the frame of the Mitochondria in Progression of Endometrial and Ovarian cancer - MiPEO study, approved by the local ethical committee. Internal review board protocols were followed for collection of samples. The study was performed on 8 patients with BOT and peritoneal implants, after collection of informed consent obtained in compliance with the Helsinki Declaration. The mean age of patients was 42.75 years (range from 23 to 81 years); all cases were diagnosed at the S. Orsola Hospital, Bologna. An alpha-numeric code (from B1 to B8) was assigned to the cases to maintain anonymity. Tumor stage was determined according to the International Federation of Gynecology and Obstetrics guidelines (FIGO) [1].

Tumors Specimens

All samples were formalin-fixed and paraffin-embedded. Haematoxylin and eosin sections were reviewed to identify paraffin blocks containing sufficient tumor cells. Paraffin blocks were cut in 10 µm sections, and 10 slides were prepared from each tissue block. Specific areas of normal tissue, BOT or peritoneal implant were marked on the different stained slides. Five unstained slides were aligned by morphology to the stained slide and corresponding areas were microscope-guided dissected with a scalpel. Selected areas were harvested from all 5 slides and placed in microfuge tubes. Microdissected areas were always scraped peripherally, to avoid contamination with other areas. Non-tumor specimens were obtained from formalin-fixed and paraffin-embedded non-metastatic lymph nodes. Histopathological diagnosis was made according to WHO criteria and the tumors were staged following the American Joint Committee on Cancer and FIGO [2].

Mitochondrial DNA Sequencing

After deparaffination, whole genomic DNA was extracted with the Mammalian Genomic DNA Miniprep Kit (Sigma) according to the manufacturer’s protocols. Sanger sequencing of the whole mitochondrial DNA was performed. Genomic DNA (5 ng) was used for amplification with a set of 46 primer pairs as previously described [3] and PCR amplification was performed using KAPA2G Fast PCR Kits (KAPA Biosystems) in a 9700 thermal cycler following the conditions given by the manufacturer. The purified PCR product (5-10 ng) was used for direct sequencing with BigDye kit version 1.1 (Thermo Fisher Scientific). Sequences were run in an ABI 3730 Genetic Analyzer automated sequencing machine. Electropherograms were analyzed with SeqScape version 2.5 software (Applied Biosystems). Mitochondrial DNA mutations detected in this first phase were confirmed using a second PCR reaction. When the latter showed the same mitochondrial DNA variant of interest, the mutation was confirmed on a second extraction of DNA from the same sample to exclude DNA contamination or sample mix-up. The somatic (informative) nature of mitochondrial mutations was ascertained by sequencing mitochondrial DNA from matched unaffected tissues.

Denaturing High Performance Liquid Chromatography (dHPLC) Analysis

An efficient detection of low-level heteroplasmies is of paramount importance since the identification of low-heteroplasmic germline mitochondrial DNA mutations in matched non-tumor samples might render some mutations non-informative. In order to establish the tumor specificity of the mutations, dHPLC analysis was performed on all mutations (with the exception of the B6 mutation occurring in the D-loop poly-C tract), which has shown to detect heteroplasmic mutations as low as 2% [4]. To this aim, PCR was performed using AmpliTaq Gold polymerase (Thermo Fisher Scientific). Primers used for the m.15570 T>C and for the m.15449 T>C in *MT-CYB* were: FW-5’- ACGAAACGGGATCAAACAAC -3’ and RV-5’- GGAGGATGGGGATTATTGCT -3. Primers used for the m.11984 T>C in *MT-ND4* were: FW-5’- CCTCGCTAACCTCGCCTTA -3’ and RV-5’- CGGTAATGATGTCGGGGTTG -3’. Primers used for the m.16189 T>C in *MT-D-loop* were: FW-5’- CGTACATTACTGCCAGCCAC -3’ and RV-5’- GGGGACGAGAAGGGATTTGA -3’ The amplification product was analyzed by WAVE Nucleic Acid Fragment Analysis System (Transgenomic, Omaha, NE, USA). Data analysis was performed as previously described [4].

Fluorescent PCR (F-PCR)

F-PCR is a sensitive technique suitable for the evaluation of heteroplasmy in difficult sequence contexts such as homopolymeric stretches, particularly when insertions/deletions are present [19]. Here F-PCR and data analyses were performed essentially as previously reported [19]. Primers used for the m.310insC in MT-D-loop were: FW-5’- CGCTTTCCACACAGACATCA -3’ and RV-5’- [Flc]TAGTATGGGAGTGGGAGGGG -3’.

Mitochondrial DNA variants analysis

Fasta files from BOT and peritoneal implants were used as input for MToolBox [5] in order to annotate mitochondrial variants and related features. The MToolBox pipeline includes several steps as read mapping, post-mapping processing, genome assembly, haplogroup prediction and variant annotation. The annotation process is mainly based on the comparison with both the two widely used rCRS and RSRS reference sequences and the recognition of alleles that are not shared with the sample-specific Macro Haplogroup Consensus Sequences [5]. The prioritization takes into account also the pathogenicity of each mutated allele, determined with different algorithms, and the nucleotide variability of each variant site; amino acid variability is also considered if the variant site is codogenic [6]. Nucleotide site-specific variability was estimated on the multi-alignment of the updated healthy genomes reported in HmtDB [7]. A training dataset of 53 mitochondrial DNA non-synonymous variants previously validated as affecting function was used to define the disease score of any non-synonymous mitochondrial DNA variants, by weighting the 6 pathogenicity predictions: MutPred [8], HumDiv- and HumVar-trained PolyPhen-2 models [9], SNPs&GO, PhD-SNP [10], and PANTHER algorithms [11]. available in the ‘patho_table’ implemented in MToolBox (https://sourceforge.net/projects/mtoolbox/). These six methods were chosen as the most widely used pathogenicity predictors to select the mitochondrial variants with a potential functional impact. Pathogenicity of a mutation, i.e. a high disease score (DS), was therefore defined when DS>0.4311, as previously calculated in [21]. Sequences of B1-B8 samples, including matched non-tumor sequences, were submitted to the public database HmtDB, list of specimens and HmtDB identifiers are reported in Additional file 2.

References:

1. Shepherd JH. Revised FIGO staging for gynaecological cancer. Br. J. Obstet. Gynaecol. 1989;96:889–92.

2. Sobin LH, Wittekind C, International Union against Cancer, editors. TNM: classification of malignant tumours. 6th ed. New York: Wiley-Liss; 2002.

3. Guerra F, Kurelac I, Cormio A, Zuntini R, Amato LB, Ceccarelli C, et al. Placing mitochondrial DNA mutations within the progression model of type I endometrial carcinoma. Hum. Mol. Genet. 2011;20:2394–405.

4. Kurelac I, Lang M, Zuntini R, Calabrese C, Simone D, Vicario S, et al. Searching for a needle in the haystack: comparing six methods to evaluate heteroplasmy in difficult sequence context. Biotechnol. Adv. 2012;30:363–71.

5. Calabrese C, Simone D, Diroma MA, Santorsola M, Gutta C, Gasparre G, et al. MToolBox: a highly automated pipeline for heteroplasmy annotation and prioritization analysis of human mitochondrial variants in high-throughput sequencing. Bioinforma. Oxf. Engl. 2014;30:3115–7.

6. Santorsola M, Calabrese C, Girolimetti G, Diroma MA, Gasparre G, Attimonelli M. A multi-parametric workflow for the prioritization of mitochondrial DNA variants of clinical interest. Hum. Genet. 2016;135:121–36.

7. Rubino F, Piredda R, Calabrese FM, Simone D, Lang M, Calabrese C, et al. HmtDB, a genomic resource for mitochondrion-based human variability studies. Nucleic Acids Res. 2012;40:D1150-1159.

8. Li B, Krishnan VG, Mort ME, Xin F, Kamati KK, Cooper DN, et al. Automated inference of molecular mechanisms of disease from amino acid substitutions. Bioinforma. Oxf. Engl. 2009;25:2744–50.

9. Adzhubei I, Jordan DM, Sunyaev SR. Predicting functional effect of human missense mutations using PolyPhen-2. Curr. Protoc. Hum. Genet. Editor. Board Jonathan Haines Al. 2013;Chapter 7:Unit7.20.

10. Capriotti E, Calabrese R, Fariselli P, Martelli PL, Altman RB, Casadio R. WS-SNPs&GO: a web server for predicting the deleterious effect of human protein variants using functional annotation. BMC Genomics. 2013;14 Suppl 3:S6.

11. Thomas PD, Kejariwal A. Coding single-nucleotide polymorphisms associated with complex vs. Mendelian disease: evolutionary evidence for differences in molecular effects. Proc. Natl. Acad. Sci. U. S. A. 2004;101:15398–403.
